# The Clinical Society

**Published:** 1896-02-22

**Authors:** 


### The Clinical Society.

### THE TREATMENT OF RUPTURED SPLEEN.

The interesting series of cases of removal of the spleen reported to the Clinical Society last Friday by Mr. Pitts and Mr. Ballance deserves the attention of all surgeons. Wandering and hypertrophied spleens have been removed often enough, and in cases in which the spleen has been involved in a wound or laceration or has been prolapsed in conjunction with such an injury, it has been removed by various surgeons, but the removal of a spleen for traumatism without any external wound, as a method of stopping a progressive and dangerous internal hæmorrhage, is almost a new proceeding. In fact the authors of the paper could only find one previous case recorded. The three cases on which the paper was founded all occurred from accident; in all there were symptoms of extensive hæmorrhage with great collapse, and the physical signs were practically identical, viz., those of fluid in the cavity of the peritoneum with marked increase of dulness in the splenic region not removable by alteration of position, whereas the dulness in the right flank passed away on placing that flank uppermost. The operation performed was as follows: An incision two inches in length was made in the middle line below the umbilicus. In each case, on reaching the peritoneum, it was found to be dark, and on incising it blood escaped freely. The nature of the case being thus made clear, an incision was made in the left linea semilunaris, from the costal cartilage downwards, large enough to admit the hand, great quantities of blood were evacuated, the layers of clot surrounding the spleen were cleared away, the spleen was drawn out of the wound, and, after its pedicle had been transfixed and tied, was cut away. The peritoneum was carefully cleared of blood by sponges, and the wound was closed. All cases recovered, but it must not therefore be thought that the removal of a healthy spleen is a small affair; for although one case, in which there was a speniculus, escaped with comparatively slight after-trouble, both the others suffered very severely about a fortnight after the loss of their spleens from changes in the blood, the red cells diminishing greatly in number and the white cells increasing, while the hæmoglobin was greatly lessened. The alterations in the blood were accompanied by great loss of strength and weight and persistent rise of temperature. It was evident that these two patients narrowly escaped death during the period preceding the full development of compensatory changes. In the discussion which followed it was suggested by some speakers that perhaps less heroic measures might have been adopted, such as the actual cautery. It was stated, however, in reply by Mr. Pitts, that this question had been discussed in the paper, that the cases described were far too urgent for any such measure; and that in cases fitted for less radical procedures, the use of deep suture of the splenic substance had been strongly recommended in the paper, but that this plan, or the employment of the iodoform gauze tampon, would not be safe if the rupture had extended into the vessels of the hilum. The patients were shown at the meeting of the society, appeared to be in robust health, and in two of them the cervical, axillary, and inguinal lymphatic glands were markedly enlarged.

